# Measuring Floodplain Inundation Using Diel Amplitude of Temperature

**DOI:** 10.3390/s20216189

**Published:** 2020-10-30

**Authors:** Jorge E. Celi, Stephen K. Hamilton

**Affiliations:** 1 Grupo de Investigación de Recursos Hídricos y Acuáticos, Universidad Regional Amazónica Ikiam, Tena 150150, Ecuador; jorge.celi@ikiam.edu.ec; 2 Department of Integrative Biology and Kellogg Biological Station, Michigan State University, Hickory Corners, MI 49060, USA; 3 Cary Institute of Ecosystem Studies, Millbrook, New York, NY 12545, USA

**Keywords:** inundation, floodplains, floods, water temperature, sensors, ecohydrology

## Abstract

Assessment of inundation patterns across large and remote floodplains is challenging and costly. Inexpensive loggers that record the damping of the diel amplitude of temperature (DAT) when submerged compared to overlying air can indirectly indicate inundation. We assessed the efficacy of this approach in tropical, subtropical, and temperate floodplains by comparing direct water level measurements using pressure transducers with the indirect indication of inundation ascertained from the DAT at the same location. The approach worked better in tropical than in subtropical and temperate floodplains. However, the relatively small DATs of air in humid and densely vegetated settings made estimation of inundation more challenging compared to the drier and less vegetated settings, where a large diel range of air temperature was markedly damped beneath the water. The indirect temperature approach must be calibrated for a particular ecosystem using direct water-level measurements to define DAT thresholds that are indicative of submergence of the sensors. Temperature provides an inexpensive indicator of duration of inundation that can be particularly useful in studies of large and remote floodplains, although the development of inexpensive sensors that directly measure submergence (e.g., by resistivity) will likely become a better option in the future.

## 1. Introduction

Floodplains subject to seasonal inundation by rivers or precipitation are globally important wetlands that support rich biodiversity and provide important ecosystem services to people [1]. Inundation is the principal driver of the ecology of floodplains, influencing diversity and phenology of plant and animal communities, biogeochemical cycles, and the interactions that humans have with these ecosystems [2]. Floodplain inundation regimes are often altered by dams and diversions on river systems, and understanding the inundation regime is crucial to manage and protect floodplains. However, floodplain inundation is often complex over space and time, which makes this task challenging and sometimes leads to oversimplifications in our understanding of the functioning of floodplain ecosystems. High spatial variability in topography and hydrological connectivity, which in turn causes variability in inundation regimes and water sources, influences floodplain plant and animal communities and ecological processes (e.g., water quality, decomposition, nutrient cycling, etc.) [3,4].

Ecohydrological studies of large and remote floodplains, which are often difficult to access during inundation, face the challenge of measuring hydrological processes, including water level fluctuations. Automated equipment (e.g., pressure transducers to measure water level) can serve this purpose well, but the relatively high cost of these units limits their application over extensive areas [5,6]. Often these units work in conjunction with a second unit that corrects measurements for barometric pressure, which can double the cost of monitoring stations if placed far apart. Another option entails direct observation and recording by local inhabitants or hired personnel [7], which in spite of being an important participatory approach is restricted to specific environments, distances, and times that people are available.

Remote sensing has often been used to map floodplain features and measure seasonal inundation regimes over extensive and remote areas [8,9]. Aerial photography or optical satellite imagery can serve to delineate inundated areas on floodplains if the vegetation canopies are open enough to see the water or the type of vegetation reflects the inundation regimen, e.g., [10]. Researchers have increasingly used a combination of optical and microwave remote sensing to measure changes in the extent of inundation over large regions [8,11,12] and radar interferometry to assess temporal changes in water level [13,14,15]. Despite the capability to acquire imagery from airborne or spaceborne sensors over broad areas, the frequency of retrieval and the cost of the imagery and its processing limit the assessment of inundation patterns over extensive areas, and very dense vegetation can impede the ability to detect inundation by remote sensing. In addition, remote sensing analysis requires ground truth information, such as water level monitoring, for calibration and validation purposes, e.g., [16], and such information is often unavailable for sparsely inhabited regions.

Less costly field approaches are therefore needed to study inundation regimes over extensive areas. Inexpensive cameras can be deployed to observe water level changes as well as vegetation phenology, e.g., [17,18], but the interpretation of images can require significant time and expertise. Commercially available environmental light sensors can be modified to measure resistivity, thereby providing an indicator of flooding, but that approach requires technical expertise, and the cost of each unit is ~US $75 [19]. That study also cites a number of previously published designs that employ modified temperature loggers but at somewhat greater cost. Ward et al. [12] employed less expensive Thermochron iButton temperature loggers [20] were employed to record changes in diel amplitude of temperature (DAT) over time as an indicator of inundation patterns in remote, episodically inundated systems in northern Australia [12]. In theory, the high specific heat capacity of water attenuates the DAT and is indicative of submergence of the sensor when compared to the larger DAT typical of the air when the sensor is out of the water. Their results suggest that this approach can be effective at detecting inundation and that temperature loggers provide a relatively inexpensive alternative to assess inundation across large remote areas. At the time of this writing temperature loggers cost much less than pressure transducers (US $35–40 vs. $500) and significantly less than modified light or temperature sensors for resistivity measurement.

In this study we assess the use of DAT records from temperature loggers to indicate inundation in contrasting floodplain environments. We evaluate temperature monitoring as an inexpensive, indirect approach to estimate the depth and duration of inundation in humid tropical floodplain forests of South America, dryland floodplains of Australia, and a temperate river and its deciduous floodplain forest of the northern United States. We show how the approach works in the different settings and discuss what environmental factors need to be considered to make the indirect temperature approach viable in floodplains.

## 2. Materials and Methods

### 2.1. Study Areas

**Figure 1:**
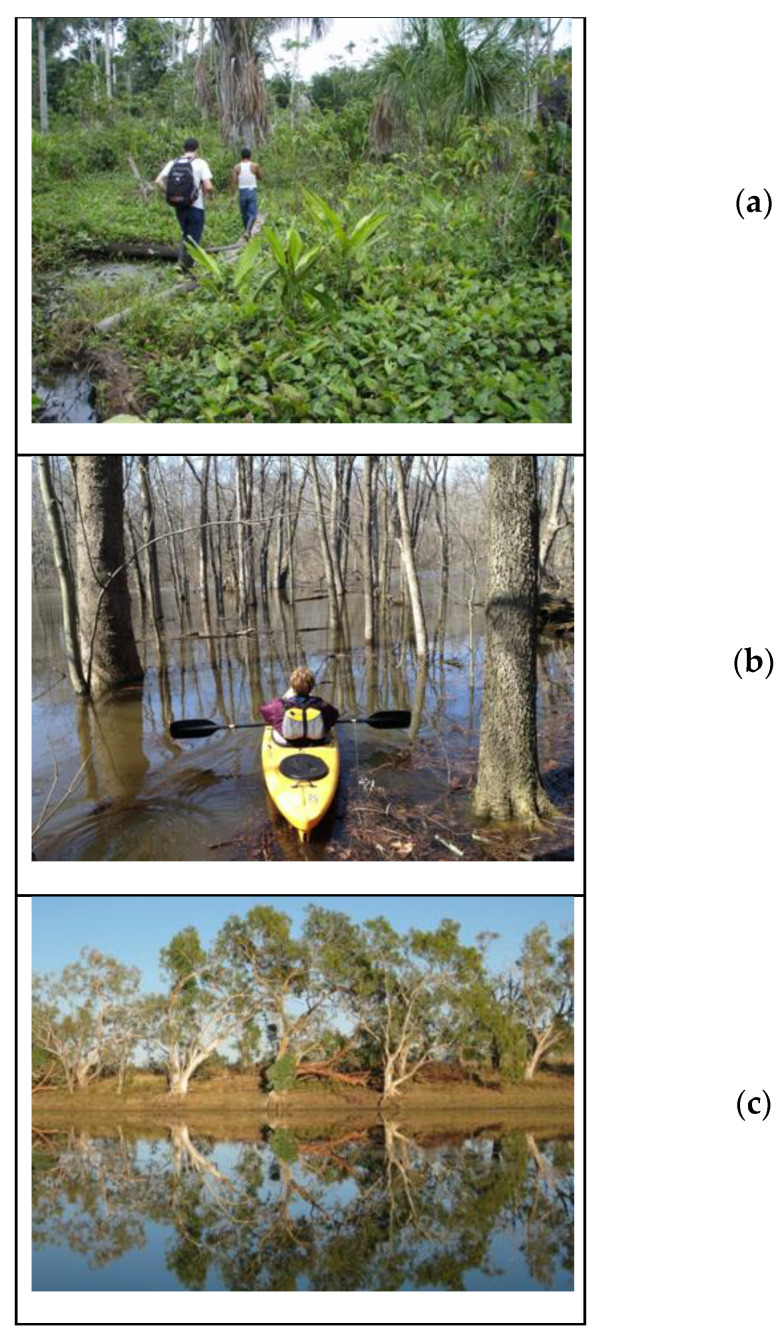
Floodplain study areas: (**a**) tropical jungle vegetation at low water in the Napo River floodplain in Ecuador; (**b**) deciduous forest flooded in early spring on the Kalamazoo River floodplain in Michigan; (**c**) river pool and floodplain in northern Australia during the dry season.

We assessed duration and depth of inundation in the floodplains of the Napo River in the humid tropics of the Amazon Basin (Ecuador and Peru), the Mitchell River in the northern wet-dry tropics of Australia (Queensland), the Moonie River in drylands of eastern Australia (Queensland), and the Kalamazoo River in the temperate humid climate of the midwestern United States (Michigan) (Table 1, Figure 1). The study areas were selected to vary in climate, hydrology, and vegetation cover, with the wettest climate and densest vegetation in the rainforest of the Napo River floodplain and the driest climate and sparsest vegetation in the Moonie River floodplain. Sensors were deployed in vertical profiles at 1–14 representative sites in each study area for periods that encompassed multiple inundation events. The Mitchell and Moonie floodplains have well-defined wet seasons during the austral winter (November–March) with inundation caused by cyclones, the Napo floodplains can experience inundation after heavy rainfall at any time of year, and the Kalamazoo River most commonly inundates its floodplain in the late winter and spring (February–May).

### 2.2. Measurements of Water Levels and Temperature

To assess the viability of the indirect temperature approach, the presence and depth of surface water were measured using pressure transducers (direct method) for comparison with temperature data (indirect method). All sensors were installed on tree trunks during low water levels and were placed to avoid direct sunlight (although most sites were shaded by vegetation canopies) and within a short distance (less than a meter). The pressure transducer (PT) and lowest temperature sensor were set at the ground level.

The PTs were either HOBO (U20 and U10) or Van Essen (TD Divers and Baros) units that recorded depth of inundation over time after correcting the records for simultaneous measurements of the local barometric pressure, which was recorded by a separate logger mounted on a nearby tree at an elevation well out of reach of flooding. The PTs recorded data six times per day at 4-h intervals with an accuracy of ±0.3–0.5 cm.

At each site, 4 to 6 temperature loggers—model 1921G or 1921H Thermochron iButtons (IBs)—were mounted at different heights above ground, including at least one above the reach of flooding (reference IB). Heights ranged from ~0.05 m to 5 m and were approximately equally spaced to encompass the expected degree of inundation of each floodplain site, as described by local guides or evident from high water marks. The distances between the sensors were therefore variable, ranging from ~10 cm to ~1 m. The sensors were sealed in 10-mil polyethylene film with minimal air and stapled to tree trunks. The IBs recorded temperature six times per day at an accuracy of ±1 °C and at 0.125 or 0.5 °C resolution.

### 2.3. Interpretation of Temperature Data to Reveal Inundation

The decrease in DAT during times when a sensor was known to be underwater (based on the PT record) relative to the DAT of the reference IB in the air above the reach of flooding was potentially specific to each site, and thus we determined site-specific DAT thresholds to calibrate our estimation of hydroperiods (i.e., time periods that sensors were underwater). These site-specific thresholds were the average of the observed DAT thresholds for each sensor at that site that was subject to submergence.

For studies across broad regions, due to variability in environmental conditions, it can be desirable to deploy a smaller number of calibration sites (i.e., colocated pressure transducers and temperature sensors) that inform the interpretation of data from a larger number of temperature monitoring sites. Such an approach requires definition of the optimal DAT threshold to apply across the broader study area. To determine whether DAT thresholds determined at sites with colocated PTs and IBs could be applied elsewhere in a particular floodplain where only IBs were deployed, we calculated the discrepancy in hydroperiod estimates that would result were an average DAT threshold used instead of a site-specific one. The discrepancy represents the difference between the “true” hydroperiod as indicated by PTs at a particular site and the indirectly estimated hydroperiod as indicated by the average DAT threshold for all sites in a particular area.

## 3. Results

### 3.1. Inundation Patterns Based on Direct Measurements

**Figure 2:**
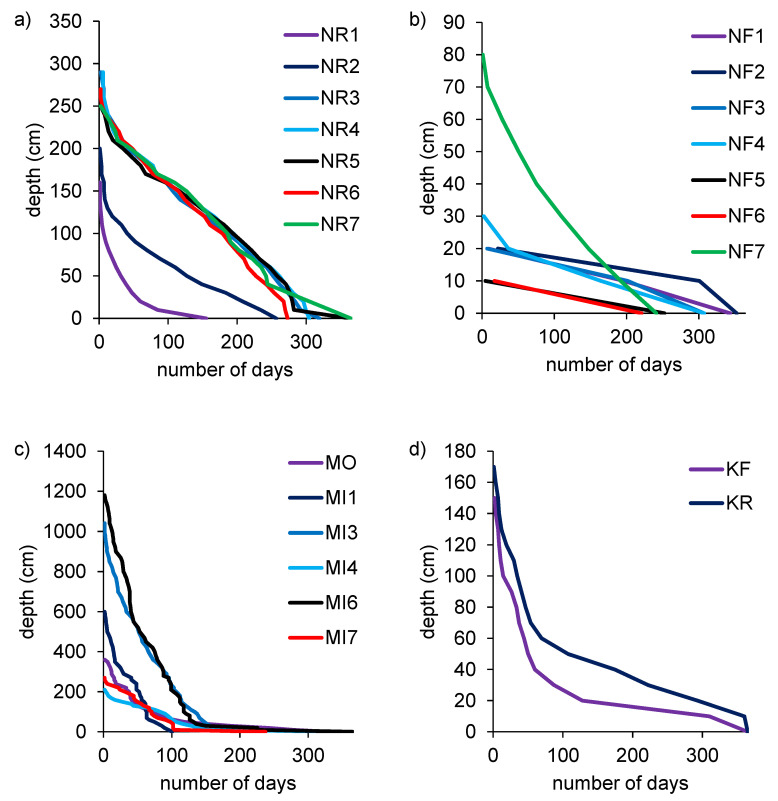
Hydroperiods based on pressure transducer measurements in the study areas: (**a**) Napo River—NR (n = 7) and (**b**) Napo floodplains—NF (n = 7) (tropical South America), (**c**) Moonie—MO (n = 1) and Mitchell rivers—MI (n = 5) (drylands of eastern and northern Australia respectively), and (**d**) Kalamazoo River—KR (n = 1 site) and floodplain—KF (n = 1) (temperate zone of the northern U.S.). Note the differences in *y*-axis scales.

The duration and depth of inundation estimated using the direct (PT-based) method varied widely among the study areas (Figure 2). Individual sites were flooded from 3.3 to 12 months of the year and to maximum depths ranging from 12 cm to almost 12 m. Although inundation events varied in frequency and duration among the three sites, in all cases multiple inundation events occurred during the period of measurement.

### 3.2. Relationship between DAT and Inundation

**Figure 3:**
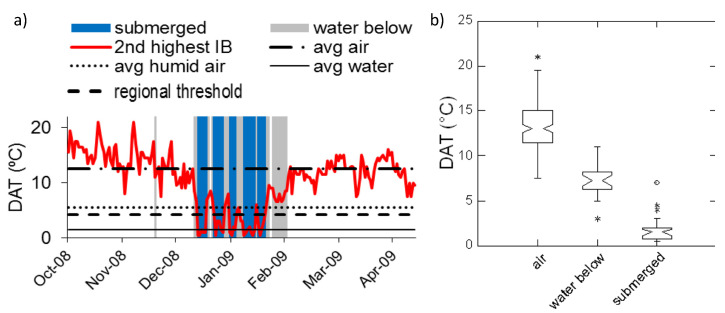
Example of the diel amplitude of temperature (DAT) observed in a tropical dryland floodplain (Rosser Creek site in the Mitchell River watershed, Australia). (**a**) Data from a representative iButtons (IB) are shown, marking periods when that sensor was underlain by standing water (gray shading) or was submerged (blue). Horizontal lines indicate average DATs for air when the site was dry (average air, abbreviated as “avg air”), when standing water was present below the sensor (“avg humid air”), and in standing water beneath the sensor (“avg water”). (**b**) DAT distributions across all sites in the study area when the sensor was in air and the floodplain was dry (“air”), in air with standing water beneath (“water below”) and submerged. Box plots show medians and interquartile ranges, and nonoverlapping notches provide an approximate indication of statistically significant differences in the medians. Asterisks indicate outliers.

**Figure 4:**
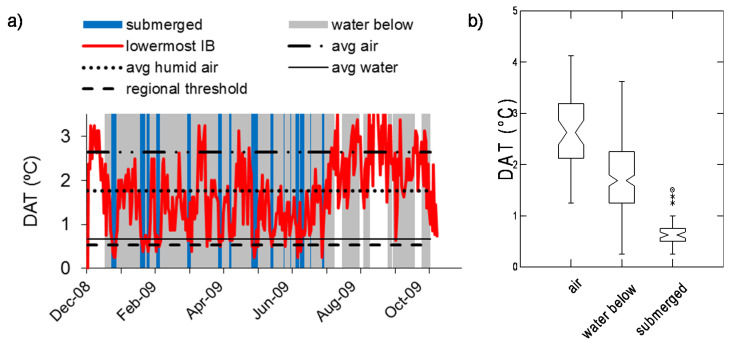
Example of the diel amplitude of temperature (DAT) observed in a humid tropical forest floodplain (Napo River at Paulacocha, Peruvian Amazon). (**a**) Data from a representative iButtons (IB) are shown, marking periods when that sensor was underlain by standing water (gray shading) or was submerged (blue). Horizontal lines indicate average DATs for air when the site was dry (average air, abbreviated as “avg air”), when standing water was present below the sensor (“avg humid air”), and in standing water beneath the sensor (“avg water”). (**b**) DAT distributions across all sites in the study area when the sensor was in air and the floodplain was dry (“air”), in air with standing water beneath (“water below”) and submerged. Box plots show medians and interquartile ranges, and nonoverlapping notches provide an approximate indication of statistically significant differences in the medians. Asterisks indicate outliers.

**Figure 5:**
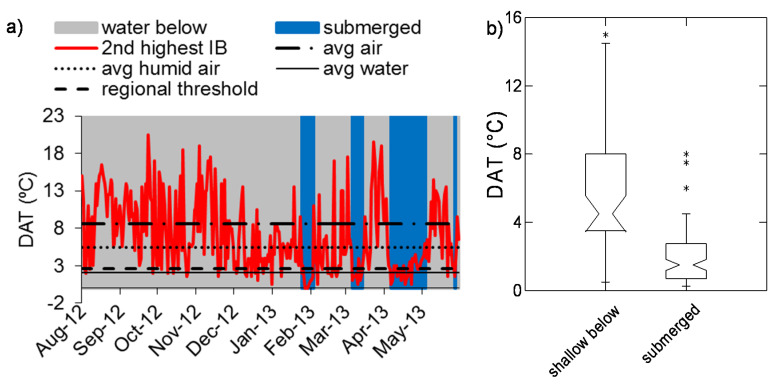
Example of the diel amplitude of temperature (DAT) observed in a humid temperate floodplain (Kalamazoo River, Michigan, USA). This site always had standing water over the ground. (**a**) Data from a representative iButtons (IB) are shown, marking periods when that sensor was underlain by standing water (gray shading) or was submerged (blue). Horizontal lines indicate average DATs for air when the site was dry (average air, abbreviated as “avg air”), when standing water was present below the sensor (“avg humid air”), and in standing water beneath the sensor (“avg water”). (**b**) DAT distributions across all sites in the study area when the sensor was in air with standing water beneath (“shallow below”) and submerged. Box plots show medians and interquartile ranges, and nonoverlapping notches provide an approximate indication of statistically significant differences in the medians. Asterisks indicate outliers.

Upon submergence we observed that sensors at all study areas displayed significantly lower DATs compared to when they were in air, as shown by examples of sites in Figure 3, Figure 4 and Figure 5. However, the difference occurred against a backdrop of high temporal variability in DATs. In addition, when there was standing water on the ground beneath the sensor, DATs at overlying sensors tended to be intermediate between DATs observed under dry and submerged conditions, as shown by the box plots of data distributions. Compared to what we observed in the seasonally flooded Australian savannas, the tropical rainforest and the temperate deciduous forest study areas showed smaller differences in DATs between sensors in air compared to periods of sensor submergence (Figure 4 and Figure 5).

### 3.3. Temperature Thresholds and Hydroperiod Estimation

**Figure 6:**
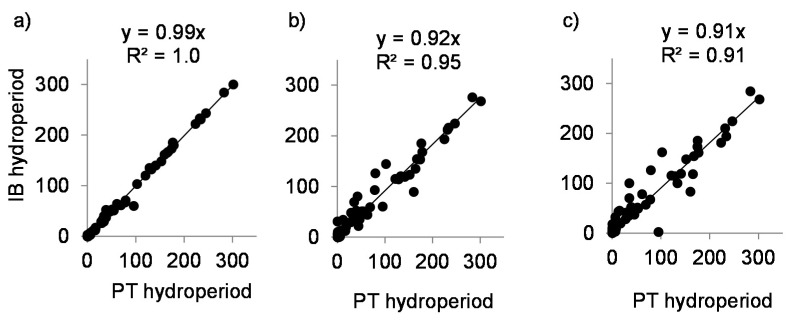
Relationship between hydroperiods for all sites combined measured directly (pressure transducers—PTs) and estimated indirectly (temperature loggers—IBs) after applying (**a**) sensor-specific, (**b**) site-specific, and (**c**) regional average DAT thresholds. In all cases the regressions are highly significant (*p* < 0.001. The 79 points represent individual IB sensors distributed across the 22 measurement sites in Table 1.

Inundation hydroperiods estimated with the indirect temperature approach using average DAT thresholds rather than sensor-specific thresholds yielded results that agreed approximately with the direct measurements (Figure 6, Table 2). However, using average DATs across all sensors in a profile at a site, or among all sites in a study area, increased the variance and resulted in a tendency for hydroperiod underestimation (negative bias averaging 8–9%, as indicated by regression fits) using the indirect method. The means of sensor-specific DAT thresholds for sites within each study area ranged from 0.67 to 5.93 °C (Table 2); DATs below these thresholds coincided with inundation of the sensors.

Within a study area, site-specific discrepancies arose because DAT thresholds varied with sensor position in the vertical profile (Table 2). Regional discrepancies were still greater, presumably reflecting site-to-site spatial variability, (i.e., degree of canopy closure by vegetation, microtopography, etc.) that would cause uncertainty when applying calibrations from a subset of locations to a broader area (Table 2). However, in spite of the discrepancies using the different DAT thresholds, the hydroperiods obtained with direct and indirect methods showed strong and significant correlations.

## 4. Discussion and Conclusions

Overall, we found that temperature could serve as an indirect indicator of inundation in all of the study areas, based on the comparisons to the direct water level measurements using pressure transducers. The accuracy and applicability of the indirect approach relied on DAT threshold determination. In spite of the effects of temporal and spatial variability, regional DAT thresholds can be applied for inundation assessment in a particular study area but at a cost of lower accuracy. The advantage of this approach is that a network of indirect temperature measurements can be deployed across large areas at a relatively low cost, while installing standard water level loggers at a smaller number of representative calibration sites.

The variability in DAT thresholds among study areas and within a particular study area presents a challenge because it necessitates a set of representative calibration sites for studies representing large areas, thereby increasing the cost of the sensor network. Unless a large number of sites are instrumented, the need for calibration sites with PTs may make the total cost higher than using the resistivity sensors described by [19], which would not require calibration against PTs. The resistivity sensors, which also record temperature, provide an unambiguous indicator of submergence, and would therefore seem to be a superior choice if a research project possesses the necessary funds and expertise. Deployment of a network of sensors in the field, whether based on temperature or resistivity, entails additional costs of field work that may make remote sensing approaches more attractive, but the use of remote sensing technologies should always include ground truth collected in the system under study for calibration and validation of the data analysis and interpretation.

In conclusion, the indirect temperature approach can serve to assess inundation across extensive, remote areas at relatively low cost compared with direct water-level sensing methods. However, attention should be paid to the environmental settings where this method is to be applied, and an initial evaluation should be conducted to assess the effects of these conditions on the relative temperatures of air and water and therefore on the prospect of success in applying this approach in a particular system. This approach may work best in dryland floodplains that are only episodically flooded and have large DATs in air due to low humidity and sparse vegetation. Constant presence of standing water below the sensors and/or high humidity under dense vegetation canopies reduce the DAT in air, making it less distinct from the DAT underwater during inundation. In addition, deep inundation provides more buffering against temperature changes than very shallow inundation, where water temperature may more closely track air temperature over the diel cycle.

## Figures and Tables

**Table 1:** Study areas in Australia, Ecuador, Peru, and the USA.

River System	Region	Latitude/Longitude Range	Biome	Climate	No. of Sites	Study Period
Mitchell	Northern Australia	15.35–16.71° S/141.72–143.41° E	Wet–dry savanna	Dry tropical	5	8 October–9 May
Moonie	Eastern Australia	28.93° S/148.74° E	Wet–dry savanna	Dry subtropical	1	7 October–8 April
Napo	Western Amazon	0.73–3.03° S/75.69–73.15° W	Rainforest	Humid tropical	14	8 December–9 December
Kalamazoo	Michigan	42.32° N/85.36° W	Deciduous forest	Temperate	2	12 August–13 June

**Table 2:** Comparison of DAT thresholds for individual sensors and means for all sites within a floodplain and resultant hydroperiod discrepancies between direct and indirect hydroperiod estimation in the study areas using DATs averaged for all sensors in a vertical profile (“Site”) and for all sensors in the floodplain study area (“Regional”). In parentheses are regression results between hydroperiods measured with the direct (PT; independent variable) and indirect (IB) methods.

	Threshold (°C)		Discrepancy (Days)	
Location	Sensor Specific	Mean of all Sites	Site	Regional
Napo floodplains	0.50–1.90	0.67 ± 0.35	4.29 ± 4.14 (r^2^ = 0.09, *p* < 0.00)	12.88 ± 10.66 (r^2^ = 0.41, *p* = 0.04)
Napo River	0.50–2.10	1.01 ± 0.35	11.08 ± 8.04 (r^2^ = 0.99, *p* < 0.00)	12.16 ± 8.47 (r^2^ = 0.99, *p* < 0.00)
Mitchell River	1.90–9.00	4.21 ± 1.38	9.97 ± 12.26 (r^2^ = 0.98, *p* < 0.00)	10.37 ± 12.16 (r^2^ = 0.88, *p* < 0.00)
Moonie River	2.60–7.60	5.93	13.33 (r^2^ = 0.99, *p* = 0.05)	--^b^
Kalamazoo River & floodplain	1.50–5.00	2.56 ± 0.65	24.75 ± 6.01 (r^2^ = 0.98, *p* < 0.00)	26.10 ± 4.38 ^c^ (r^2^ = 0.97, *p* < 0.00)
**All sites**	0.50–9.00	2.00 ± 1.79	10.29 ± 9.38 (r^2^ = 0.92, *p* < 0.00)	13.32 ± 9.97 (r^2^ = 0.91, *p* < 0.00)

^a^ The DAT thresholds are summarized as means and standard deviations of the site thresholds (means for the study areas) employed to estimate regional discrepancies. Site discrepancies were estimated using the mean of the sensor-specific thresholds employed in each site. ^b^ Only one site was monitored in the Moonie River. ^c^ Only two sites were monitored in the Kalamazoo River system.

## References

[1] Hamilton S.K., Likens G.E. (2009). Flood Plains. Encyclopedia of Inland Waters.

[2] Junk W.J., Bayley P.B., Sparks R.E. (1989). The flood pulse concept in river-floodplain-systems. Can. Spec. Publ. Fish. Aquat. Sci..

[3] Junk W.J., Wantzen K.M., Nunes da Cunha C., da Silva C.J. (2011). Ecology, biodiversity and sustainable management of the Pantanal: A synthesis. The Pantanal of Mato Grosso: Ecology, Biodiversity and Sustainable Management of a Large Neotropical Seasonal Wetland.

[4] Lewis J.W.M., Hamilton S.K., Lasi M.A., Rodríguez M.A., Saunders J.F. (2000). Ecological determinism on the Orinoco floodplain. BioScience.

[5] Conly F.M., Su M., Kamp G., Millar J.J. (2004). A practical approach to monitoring water levels in Prairie wetlands. Wetlands.

[6] Greswell R., Ellis P., Cuthbert M.O., White R., Durand V. (2009). The design and application of an inexpensive pressure monitoring system for shallow water level measurement, tensiometry and piezometry. J. Hydrol..

[7] Danielsen F., Burgess N.D., Balmford A., Donald P.F., Funder M., Jones J.P.G., Alviola P., Balete D.S., Blomley T., Brashares J. (2009). Local Participation in Natural Resource Monitoring: A Characterization of Approaches. Conserv. Biol..

[8] Alsdorf D.E., Rodríguez E., Lettenmaier D.P. (2007). Measuring surface water from space. Rev. Geophys..

[9] Melack J.M., Hess L.L., Junk W.J., Piedade M., Wittmann F., Schöngart J., Parolin P. (2010). Remote Sensing of the Distribution and Extent of Wetlands in the Amazon Basin. Amazonian Floodplain Forests: Ecophysiology, Ecology, Biodiversity and Sustainable Management.

[10] Gennai M., Carnicelli S., Dell’Olmo L., Gabellini A., Giunti M., Lazzaro L., Lucchesi F., Monacci F., Viciani D., Foggi B. (2020). The Floodplain Woods of Tuscany. J. Maps.

[11] Lee H., Beighley R.E., Alsdorf D., Jung H.C., Shum C., Duan J., Guo J., Yamazaki D., Andreadis K. (2011). Characterization of terrestrial water dynamics in the Congo Basin using GRACE and satellite radar altimetry. Remote Sens. Environ..

[12] Ward D.P., Hamilton S.K., Jardine T.D., Pettit N.E., Tews E.K., Olley J.M., Bunn S.E. (2012). Assessing the seasonal dynamics of inundation, turbidity, and aquatic vegetation in the Australian wet-dry tropics using optical remote sensing. Ecohydrology.

[13] Alsdorf D., Birkett C., Dunne T., Melack J., Hess L. (2001). Water level changes in a large Amazon lake measured with spaceborne radar interferometry and altimetry. Geophys. Res. Lett..

[14] Jung H.C., Hamski J., Durand M.T., Alsdorf D., Hossain F., Lee H., Hossain A.K.M.A., Hasan K., Khan A.S., Hoque A.Z. (2010). Characterization of complex fluvial systems using remote sensing of spatial and temporal water level variations in the Amazon, Congo, and Brahmaputra Rivers. Earth Surf. Process. Landf..

[15] Poncos V., Teleaga D., Bondar C., Oaie G. (2013). A new insight on the water level dynamics of the Danube Delta using a high spatial density of SAR measurements. J. Hydrol..

[16] Costache R., Popa M.C., Tien Bui D., Diaconu D.C., Ciubotaru N., Minea G., Pham Q.B. (2020). Spatial predicting of flood potential areas using novel hybridizations of fuzzy decision-making, bivariate statistics, and machine learning. J. Hydrol..

[17] Pettit N.E., Jardine T.D., Hamilton S.K., Sinnamon V., Valdez D., Davies P.M., Douglas M.M., Bunn S.E. (2012). Seasonal changes in water quality and macrophytes and the impact of cattle on tropical floodplain waterholes. Mar. Freshw. Res..

[18] Nezval O., Krejza J., Světlík J., Šigut L., Horáček P. (2020). Comparison of traditional ground-based observations and digital remote sensing of phenological transitions in a floodplain forest. Agric. For. Meteorol..

[19] Chapin T.P., Todd A.S., Zeigler M.P. (2014). Robust, low-cost data loggers for stream temperature, flow intermittency, and relative conductivity monitoring. Water Resour. Res..

[20] Hubbart J., Link T., Campbell C., Cobos D. (2005). Evaluation of a low-cost temperature measurement system for environmental applications. Hydrol. Process..

